# Considerations and Software for Successful Immune Cell Deconvolution Using Proteomics Data

**DOI:** 10.1021/acs.jproteome.4c00868

**Published:** 2025-07-14

**Authors:** Måns Zamore, Sergio Mosquim Junior, Sebastian L. Andree, Can Altunbulakli, Malin Lindstedt, Fredrik Levander

**Affiliations:** † Department of Immunotechnology, 5193 Lund University, SE-22363 Lund, Sweden; ‡ National Bioinformatics Infrastructure Sweden, Science for Life Laboratory, Lund University, SE-22363 Lund, Sweden

**Keywords:** proteomics, cell-type deconvolution, immune cells, LC-MS/MS, immune infiltration

## Abstract

Inferring the cell-type composition of bulk samples can provide biological insight. While bulk transcriptomics data has been extensively used for this purpose, the use of proteomics data has remained unexplored until recently. This study evaluates computational approaches for estimating immune cell composition using bulk sample proteomics data. Leveraging defined immune cell populations and simulated mixtures, we assess the impact of preprocessing methods and software tools on cell deconvolution outcomes. Our findings demonstrate the feasibility of using proteomics data for cell-type deconvolution, with Pearson correlations for estimated proportions in simulated sample mixtures above 0.9 when employing optimal missing value imputation and reference matrix generation parameters. We further provide an R package, proteoDeconv, to facilitate the preprocessing of proteomics data for deconvolution and parsing of results. This study highlights the feasibility of using proteomics for analyzing cell-type composition in biological samples.

## Graphical abstract

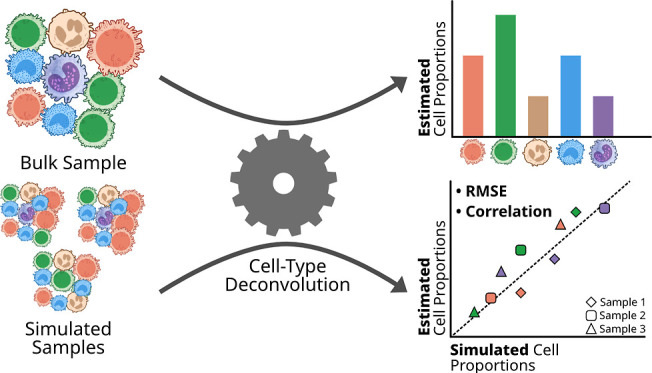

## Introduction

The type, location and level of tumor-infiltrating immune cells have been shown to have prognostic value for many cancer types.^1–7^ Aspects of immune infiltration in the tumor microenvironment may be utilized in clinical decision-making, for instance for stratification of patients into treatment groups. Several direct methods for measuring immune cell infiltration exist, for example flow cytometry and immunohistochemistry. Alternatively, immune cell infiltration has been successfully estimated using bulk transcriptomics coupled with deconvolution algorithms.^8^ An advantage of such a strategy is that the cell composition of a sample can be estimated from a transcriptomics sample, which is used also for measuring other markers in parallel. However, as cell types can be defined by the levels of many protein markers, deconvolution is complex and the absolute levels of proteins are difficult to estimate based on transcript levels alone. With proteomics measuring the actual end product—proteins—rather than just the transcript, one could potentially get more accurate information about immune cell infiltration than with transcriptomics. However, the use of proteomics coupled with deconvolution algorithms has been largely unexplored until recently.

Available deconvolution tools have mainly been developed for transcriptomics data and can be broadly categorized into two main approaches: marker gene-based and reference-based methods.^8^ Marker gene-based approaches rely on specific genes associated with each cell type. Models quantify cell types independently by analyzing the expression of marker genes in heterogeneous samples. Reference-based methods, on the other hand, treat the problem as a system of equations. They describe gene expression in a sample as a weighted sum of expression profiles from different cell types. By solving this inverse problem, cell-type fractions can be inferred (Figure 1). Deconvolution tools can also be classified as partial or complete, depending on if only the cell types that are included in the signature matrix are quantified (partial deconvolution) or if the algorithm attempts to quantify all cell types in the sample (complete deconvolution). Popular algorithms for immune deconvolution include CIBERSORTx^9^ and EPIC.^10^ They have been extensively evaluated with transcriptomics data.^11,12^ The developments in recent years (so-called second-generation immune deconvolution) have focused on algorithms that generate signatures from single-cell RNA sequencing (scRNA-seq).^8^

**Figure 1:**
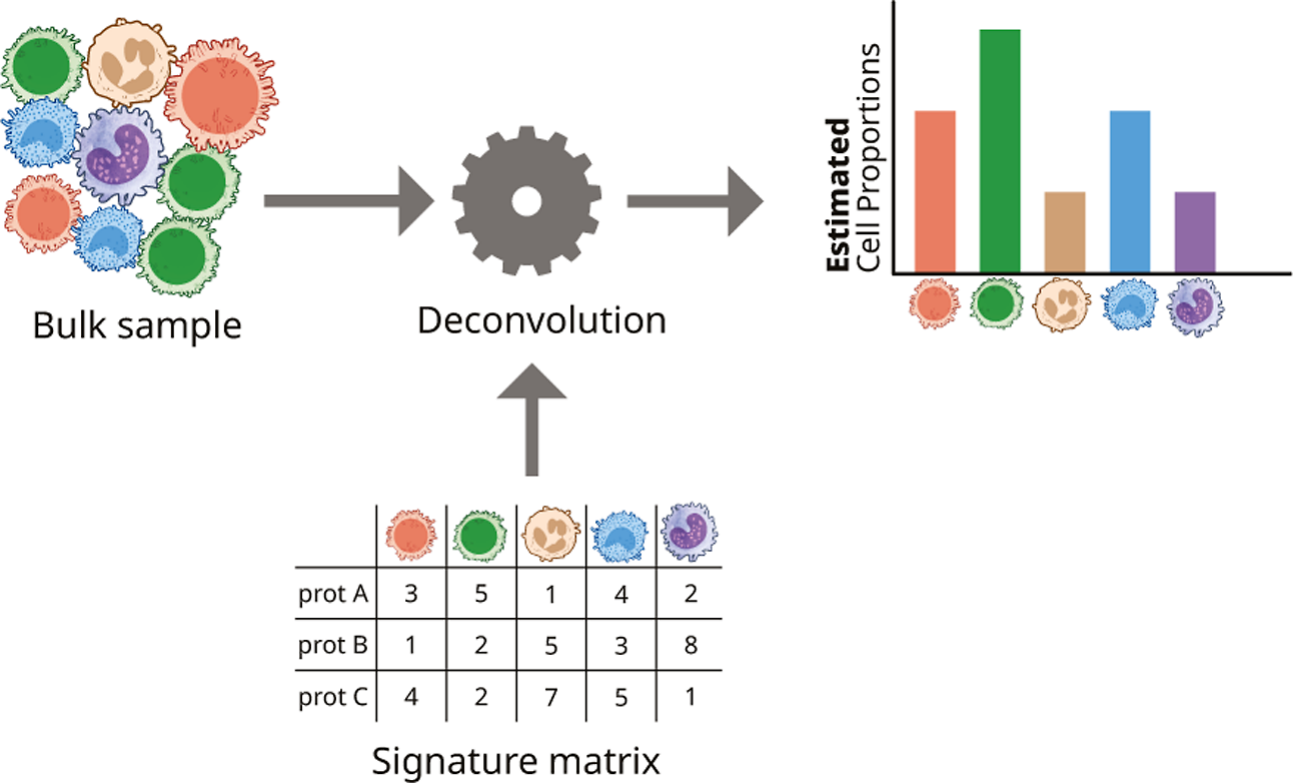
Schematic figure showing the principle behind reference-based immune deconvolution.

Deconvolution tools have also been developed for other types of omics than transcriptomics, such as DNA methylation^13,14^ and chromatin accessibility.^15,16^ Proteomics for deconvolution has received some attention recently.^17–22^ Notably, two different algorithms have been developed: BayesDeBulk^18^ and scpDeconv.^22^ Neither of these algorithms has been evaluated in independent benchmarks. The only proteome-based assessment of deconvolution algorithms so far is the recent Decomprolute benchmarking platform.^20^ The effect of various preprocessing methods has however not been investigated with Decomprolute.

In this study, we explore various approaches to immune deconvolution with proteomics data and the impact of different steps. Preprocessing steps, including normalization, imputation, and gene symbol handling, are discussed and recommendations are provided. We evaluate deconvolution performance on both pure immune cell samples and artificial simulated mixtures. Multiple deconvolution algorithms are benchmarked, including BayesDeBulk,^18^ CIBERSORTx,^9^ CIBERSORT,^23^ and EPIC.^24^ We also present an R package, proteoDeconv, which facilitates pre- and postprocessing for immune deconvolution with proteomics data.

## Experimental Section

### Defined Immune Cell Data and Mixtures

#### Immune Cell Preparation for Proteomic Analysis

PBMCs were isolated with Ficoll–Paque PLUS (Cytiva, Uppsala, Sweden, 17144003) density centrifugation and collection of the lymphocyte layer in PBS-EDTA (Invitrogen, Grand Island, NY, USA, 15575-038). Isolated PBMCs were rested in RPMI media (Cytiva, South Logan, UT, SH30096.01) supplemented with 10% FBS (Gibco, Paisley, UK, 10270-106) and 2 mM l-glutamine (Cytiva, SH30034.01). Isolated cells were counted with 1:9 vol/vol trypan blue (Gibco, Green Island, NY, USA, 15250-061) on a Luna Counter FL system (Logos Biosystems, Anyang-si, Gyeonggi-do, South Korea, L20001), ensuring a cell viability higher than 90%. The PBMC replicates for MS analysis were prepared by transferring 1 μL of the cell suspension (3.92 × 10^7^ cells/ml) into 79 μL PBS and splitting into four 20 μL fractions.

Isolated PBMCs were stained with viability staining (BD Horizon Fixable Viability Stain 620, BD Biosciences, 564996), Fc receptors were blocked (ChromPure Mouse IgG, Jackson ImmunoResearch Laboratories INC., Ely, Cambridgeshire, UK 015-000-003) and then subsequently stained with our flow cytometry panel. The antibody panel consists of: CD3-APC (Life Technologies, MHDC0305), CD19-RPE (Dako, R0808), CD56-BV605 (BioLegend, 362537), CD8-PE/Cy7 (BD Pharmingen, 557746), CD14-FITC (Life Technologies, 2300712), CD27-BV510 (BD Horizon, 563092), CD20-PerCP/Cy5.5 (BD Pharmingen, 560736), CD16-BV786 (BD Horizon, 563690), HLA-DR-BV711 (BD Horizon, 563696). Acquisition and sorting of target cell populations were performed on BD FACSAria IIu (BD Biosciences, San Jose, CA) cell sorter. B-cells, naïve B-cells, T-cells, cytotoxic T-cells, Monocytes and activated NK cells were sorted approximately 10,000 cells/target population with a 100 μm nozzle in 4-way-purity mode. Mixes of 50% cytotoxic T-cells and 50% monocytes were also acquired. 100 μL 2% SDS (pH7) was added to each cell sample and the samples were heated to 95 °C for 7 min. 100 μL 2% SDS 20 mM CAA 10 mM TCEP 0.1 M Tris was then added and samples were left standing at room temperature before freezing at −80 °C followed by thawing and sonication.

Samples were prepared for mass spectrometry using S-Trap (Protifi, Fairpoint, NY, USA, C02-micro-80), according to the manufacturer’s instructions with overnight LysC and trypsin (1:9) digestion. Peptide eluates were dried using a SpeedVac Concentrator (Thermo Fisher Scientific, Asheville, NC, USA, Savant SPD131DDA).

#### Liquid Chromatography–Mass Spectrometry Data Acquisition

Evotips (Evosep) were loaded with peptides resuspended in 0.1% formic acid according to the manufacturer’s instructions and were sequentially loaded on an Evosep One liquid chromatography system with a Picofrit 15 cm column of 360 μm OD × 75 μm ID (CoAnn Technologies LLC, Richland, WA, USA, ICT36007515F-50-5) self-packed in-house with 1.9 μm C18 (Dr.Maisch GmbH, Ammerbuch Germany, r119.aq.0003) coupled to a Q Exactive HF-X (Thermo Fisher Scientific, Waltham, MA, USA) mass spectrometer. The peptides were separated using the standard 58 min Whisper method (Whisper 20 SPD method) with a column temperature at 40 °C and the spectra were acquired in data-independent acquisition (DIA) mode. The DIA method used a normalized collision energy of 27 with automatic injection time. Data acquisition was between 4 and 58 min in positive ion mode. MS1 spectra were collected at a target resolution of 60000 with automatic gain control (AGC) target value 3 × 10^6^ and 55 ms maximum injection time in the scan range 395–1005 *m*/*z* in centroid mode. DIA MS2 spectra were acquired with 15,000 resolution and 1 × 10^6^ AGC target value and automatic maximum injection time, with 50 loop count and 12 *m*/*z* isolation window with a normalized collision energy of 27. The DIA inclusion list contained 101 staggered windows between 400 and 1000 *m*/*z* according to the 12 *m*/*z* staggered window method suggested by Pino et al.^25^

### DIA Data Processing

The proteomics raw data underwent conversion to mzML format using vendor peak-picking and demultiplexing via MSconvert v.3.0.21266-1f16dae8 ^26^ and was subsequently processed with DIA-NN version 1.8.1.^27^ In DIA-NN, library-free mode was employed, utilizing the UniProt human FASTA database from 2022–08–11 with common contaminants added as the input.^28^ Precursors with charge states ranging from 1 to 4, peptide lengths between 7 and 30, and peptide *m*/*z* values from 300 to 1800 were considered. Cysteine carbamidomethylation was set as a fixed modification, and no additional variable modifications were included. Quantification utilized “robust LC (high precision)” settings, and mass accuracy was automatically set. All proteomics data have been deposited but the NK cells were excluded from downstream analyses due to that only two replicates were available.

#### Immune Cell Reference Proteome

Data from Rieckmann et al.^29^ was used as additional reference data for the immune cell proteome. The protein group file was retrieved from ProteomeXchange repository PXD004352.^30^ Activated and steady-state cells were grouped together for all analyses, and erythrocytes and thrombocytes were excluded.

#### Simulated Mixtures

In addition to experimentally generated 50–50 mixtures of two immune cell types (CD8+ T cells and monocytes), hundreds of in silico mixtures were generated by combining randomly selected replicate samples of pure cell-type proteomes in varying fractions from 0 to 1. These mixtures were generated using the immune cell reference proteome samples from Rieckmann et al.^29^ Simulations were conducted using the SimBu package, originally developed for transcriptomics data^31^—without applying a scaling factor. Following simulation, the resulting expression matrices were subjected to deconvolution, and the estimated cell-type proportions were compared to the known simulated proportions. Pearson correlation coefficients and Root Mean Squared Errors (RMSE) were calculated for each tested cell type, and their mean values across all cell types are reported to assess deconvolution performance.

#### Signature Matrices

Custom proteomics-derived signature matrices were generated using immune cell data from Rieckmann et al.^29^ as the reference. The original 28 cell types were consolidated into seven broader groups for signature matrix generation: CD8+ T cells, CD4+ T cells, Dendritic cells, Monocytes, B cells, NK cells, and Granulocytes. These custom signature matrices were constructed using the CIBERSORTx Docker image, executed via the proteoDeconv package, with a set of optimized parameters—deviating settings from the defaults are specified in the text: the minimum and maximum numbers of genes per cell type (G.min and G.max) were set to 200 and 400, a stringent *q*-value threshold of 0.01 was applied for differential expression, while nonhematopoietic genes were retained (filter = FALSE). When comparing signature matrices from scRNA seq and proteomics, the matrix generation was reduced to the following five cell types: CD8+ T cells, CD4+ T cells, Monocytes, B cells and NK cells, with the scRNA data derived from the CIBERSORTx Web site (data set: NSCLC PBMCs Single Cell RNA-Seq). For BayesDeBulk, markers were identified using limma-based pairwise comparisons, selecting proteins with expression >1000 and at least 3-fold higher expression in the target cell type relative to others, starting from the same protein-based reference matrix as the other methods.

### Data Analysis

Data processing and analysis were conducted in R version 4.4.2 ^32^ using Posit’s Positron as IDE. When required, missing values were imputed using MsCoreUtils version 1.16.0.^33^ HGNC gene symbols were updated using HGNChelper version 0.8.14.^34^ Data were normalized with cyclic loess normalization or quantile normalization when applicable using limma version 3.60.0,^35^ and with vsn using the vsn package version 3.70.0.^36^ Following normalization, data were exponentiated to a linear scale, and each sample was scaled to a total intensity of 1 × 10^6^, analogous to the Transcript Per Million (TPM) approach in RNA-seq pipelines. CIBERSORT,^23^ EPIC,^10^ BayesDeBulk,^18^ and CIBERSORTx^9^ were executed via proteoDeconv, which internally calls their respective R packages or the Docker image, as appropriate. All processing steps were implemented in targets for reproducibility,^37^ while renv ensured a consistent computational environment. The full pipeline is available on GitHub: https://github.com/ComputationalProteomics/proteoDeconv-manuscript.

### proteoDeconv R Package

An R package was developed to facilitate immune deconvolution with proteomics data. The package includes functions for preprocessing data, updating HGNC symbols, imputing missing values, running deconvolution algorithms, simulating data, and generating signature matrices. The R package proteoDeconv is available on GitHub: https://github.com/ComputationalProteomics/proteoDeconv.

## Results

Given the potential of bulk proteomics data for cell deconvolution, we aimed to evaluate the feasibility of applying deconvolution methods originally developed for transcriptomics and to investigate how different data processing strategies affect the outcome. Numerous deconvolution algorithms have been developed for transcriptomic data analysis; among the most commonly utilized are CIBERSORT, CIBERSORTx, and EPIC, all of which are tested in our framework. Other algorithms, such as ESTIMATE,^38^ ConsensusTME,^39^ quanTIseq,^6^ MCP-counter,^40^ and TIMER,^41^ were not tested due to their limited cell type resolution or incompatibility with custom signature matrices. For proteomics-based deconvolution, two algorithms exist: BayesDeBulk (included in our framework) and scpDeconv. However, we could not test scpDeconv due to the absence of suitable single-cell proteomics immune cell data sets.

To investigate the feasibility of using these algorithms on proteomics data, we first evaluated them on a data set of pure immune cells developed by Rieckmann et al.,^29^ with a signature matrix generated from the same data set. As illustrated in Figure 2, CIBERSORT and CIBERSORTx yield identical, well-performing results. BayesDeBulk also performs well, while EPIC performs poorly. To further test the ability of the algorithms to deconvolute mixtures, we performed tests using simulated mixing of different pure immune cell samples at random proportions in 100 combinations and correlated the fractions estimated by the algorithms to the expected values. Both correlation and RMSE are important metrics in this context: correlation measures how well the estimated fractions follow the trend of the expected values, reflecting the algorithm’s ability to capture relative differences between samples, while RMSE quantifies the average magnitude of the errors, providing insight into the absolute accuracy of the estimates. The results of the simulations are provided in Table S1. Using the simulated data with the different algorithms, CIBERSORT and CIBERSORTx each had a Pearson correlation of about 0.91 and an RMSE of approximately 0.04. BayesDeBulk had a correlation of 0.76 and an RMSE of 0.07. EPIC performed worse in this case, with a correlation of 0.68 and an RMSE of 0.10. Based on these results, we continued to analyze the influence of different parameters on deconvolution results of different data sets using the CIBERSORT algorithm, as it performed well on these data and is well-regarded for transcriptomics.

**Figure 2:**
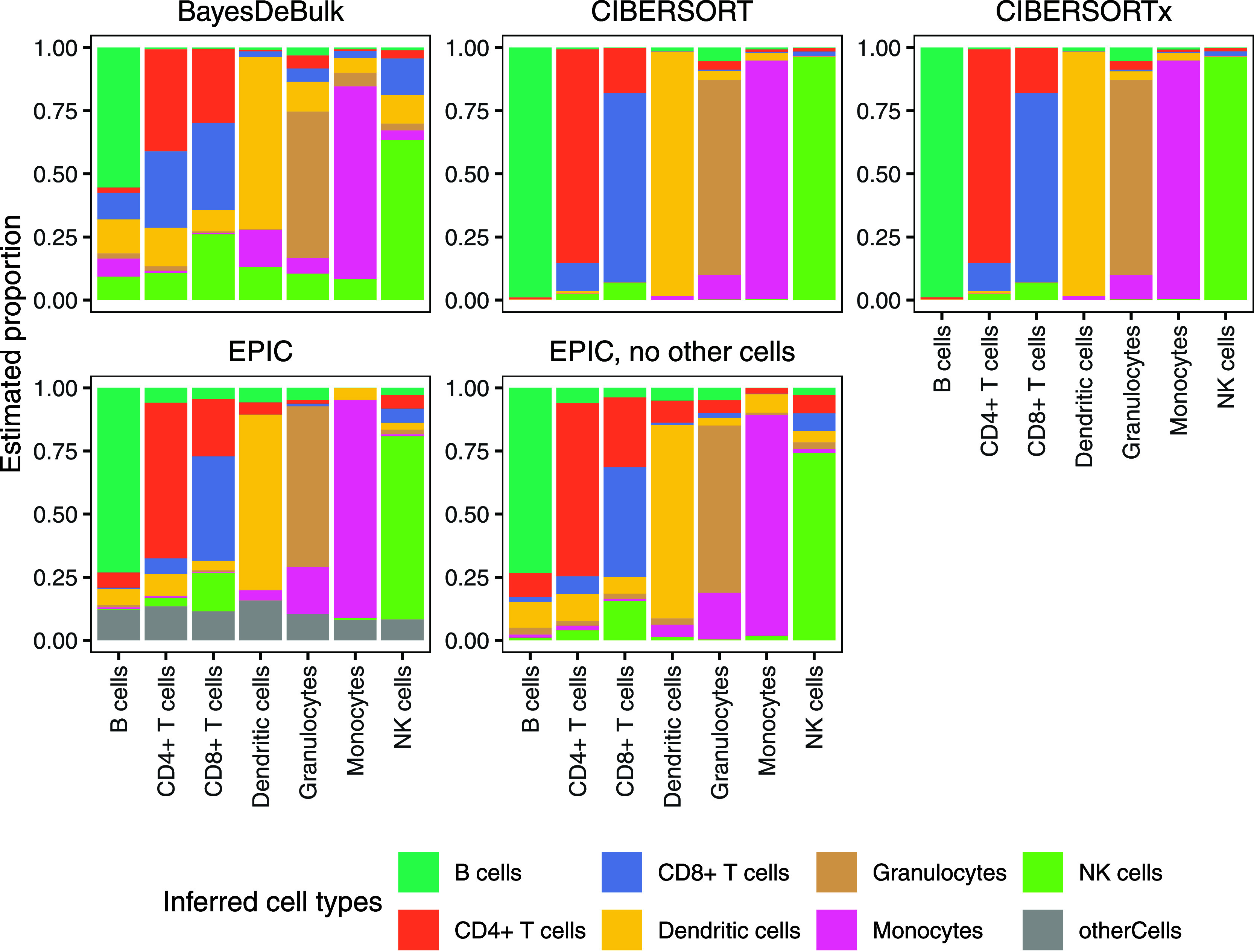
Comparison of BayesDeBulk, CIBERSORT, CIBERSORTx, and EPIC using the same DDA-based immune cell samples that served as reference for the signature matrix derived from the Rieckmann et al.^29^ data set. Each bar shows the estimated cell-type proportions for pure samples that contain only one expected cell type.

One reason why proteomics-based deconvolution algorithms have not been developed may be that more data are needed to evaluate them, as gold standard data sets of immune cell mixtures are missing. We therefore acquired new proteomics data for different immune cell types (B-cells, Naïve B-cells, T-cells, Cytotoxic T-cells, Monocytes and activated NK cells) derived from peripheral blood mononuclear cells (PBMCs). We also generated defined mixtures of cells using fluorescence-activated cell sorting that could be used to evaluate deconvolution algorithms. The data were acquired using data-independent acquisition (DIA) to reflect current state-of-the-art, to a depth of about 2000 proteins per cell type. These data were then used along with the Rieckmann data to evaluate the effects of different data processing parameters on deconvolution outcome, before testing the effects of different signature matrices.

Several challenges exist with inputting proteomics data into deconvolution tools originally developed for transcriptomics data. First, there is the problem of how to handle ambiguously identified proteins, that is protein groups, as the deconvolution algorithms do not accept protein groups. One solution is to simply select the first protein listed in each protein group. Another solution is to let the search engine (for example DIA-NN) produce a protein matrix that does not contain protein groups but single protein or gene identifiers for each entry. The difference between these two approaches on our reference data using CIBERSORT can be seen in Figure S1, visualizing that the protein grouping approach had small effects on the deconvolution outcome. Furthermore, when performing simulations using either approach, the difference is small, and it varies between data sets which method is best (Table S1).

The second problem is that there may be duplicate occurrences of proteins in proteome data, especially after reducing the protein groups to single proteins. To handle this, the protein with the highest median intensity may be chosen over the other(s) (denoted by the slice method). Another possible approach is to merge the intensities of all occurrences of a protein into one (using summarization, denoted the merge method). The deconvolution performance resulting from these two approaches is compared in Figure S1. Also in this case, the differences between the two methods were small, and it varies between data sets which approach is best.

### Normalization and Imputation

It has previously been found that deconvolution algorithms perform better with data in linear space, that is not log-transformed,^42^ and that normalizations generally disrupt the deconvolution performance.^12^ We tested the effect of applying different normalizations (Figure 3) and found that normalization indeed can have a detrimental effect on the deconvolution performance. With simulations of the Rieckmann data, the Pearson correlation for no normalization is 0.91 and the RMSE is 0.04. When normalizing with cyclic loess normalization for example, despite subsequently back-transforming the data to a linear scale, the correlation ends up lower at 0.80 and the RMSE at 0.08.

**Figure 3:**
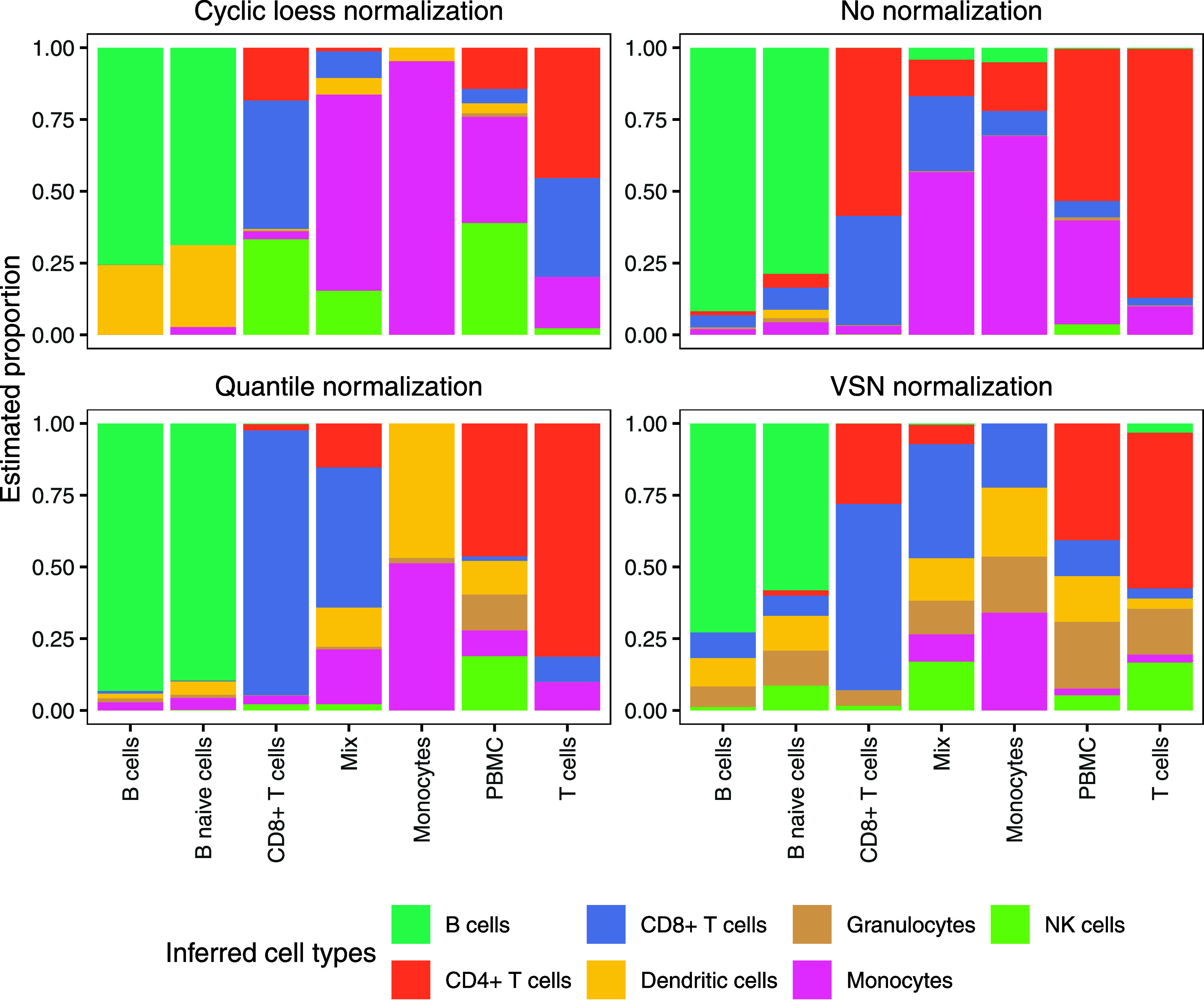
Comparison of different normalization strategies in CIBERSORT-based deconvolution of DIA immune cell samples using the Rieckmann-derived signature matrix. The mix samples contained an equal number of CD8+ T cells and monocytes, and PBMC indicates crude PBMCs. For each comparison, the normalization method was applied to both the proteome data used for the signature matrix and to the samples being deconvoluted.

Another potential issue with proteomics data is missing values and how they should be handled. Missing values are common in proteomics data, and none of the deconvolution algorithms tested in this work tolerate any missing values. Thereby the missing data need to be imputed. Several approaches to imputation exist, including Random Forest imputation (RF), minimum value imputation and standard distribution imputation. Multiple imputation methods were compared, with results indicating that a conservative imputation method with minimum value imputation works better than for example RF imputation (Figure 4). Furthermore, we also evaluated the effect of imputation on the proteomics immune cell data from Rieckmann et al.^29^ (Figure 4). For all cell types, the inferred cell type corresponds better to the actual cell type when using minimum-value imputed data, further highlighting that this conservative strategy was beneficial for the deconvolution of this data set. This finding is also reiterated by simulations of the Rieckmann data: the Pearson correlation and RMSE indicates better performance with lowest-value imputation (0.91 and 0.04, respectively) than with either kNN (0.82 and 0.06) or RF imputation (0.85 and 0.06).

**Figure 4:**
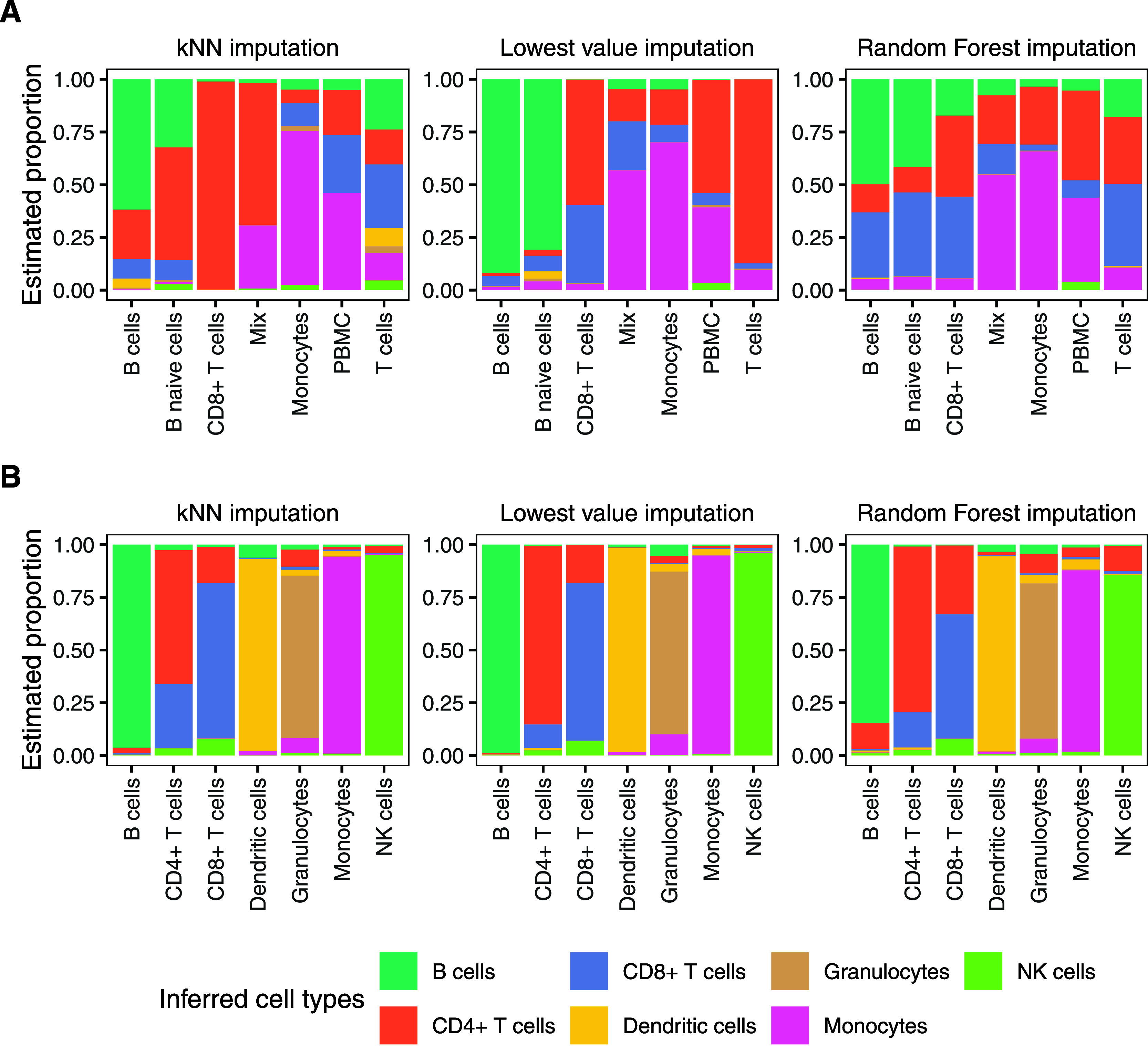
Imputation strategies for missing values in immune cell data using the Rieckmann-derived signature matrix. (A) Comparison using this study’s collected DIA data as data source. The mix samples contained an equal number of CD8+ T cells and monocytes, and PBMC indicates crude PBMCs. (B) Comparison of imputation methods as in A but with the Rieckmann et al. DDA data and the cell types in the signature matrix.

### Different Reference Matrices

Transcriptomics-derived signature matrices have been applied for immune deconvolution of proteomics data,^17^ but recent findings suggest that proteomics-derived signature matrices may be more appropriate for proteomics data.^20^ As has been shown in several studies, the correlation over samples between proteome and transcriptome data is moderate - with median Pearson/Spearman correlations around 0.4–0.5 for high-quality data.^43–45^ We therefore hypothesized that a proteome-derived signature matrix would increase the deconvolution performance. Furthermore, with immune infiltration estimates typically being dependent on protein markers (for example in the case of immunohistochemistry or cell sorting), deconvolution based on proteins will be more directly comparable. As shown in Figure 5, the proteome-derived signature matrix outperforms the single-cell RNA-sequencing-derived signature matrix in deconvolution performance, despite both being constructed from the same five cell types. Also in simulations with the Rieckmann data, the proteome-derived signature performs better with a correlation of 0.96 and RMSE of 0.05, compared to the scRNA-seq signature which yields a correlation of 0.85 and RMSE of 0.12.

**Figure 5:**
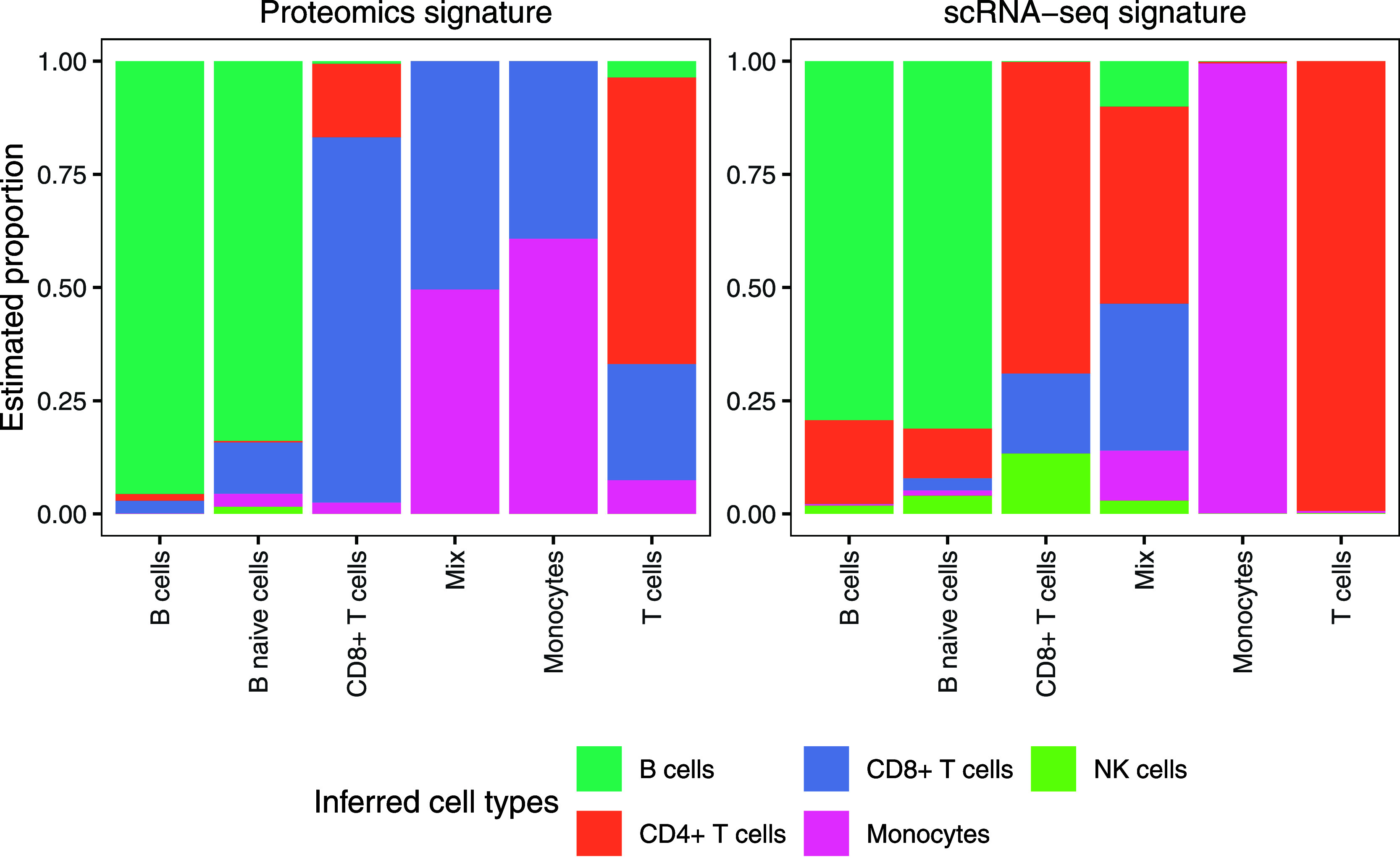
Comparison of DIA-based immune cell samples deconvoluted with a proteome-derived signature matrix (derived from the Rieckmann et al. data set) versus a single-cell RNA-sequencing-derived signature matrix. To make a fair comparison, both signatures were made using the same five cell types, with the same signature generation parameters. The mix samples contained an equal number of CD8+ T cells and monocytes.

### Parameters for Generating Reference Matrices

With transcriptomics data, the choice of the signature matrix has a substantial effect on deconvolution results, as it defines the cell-type-specific expression profiles used to estimate cellular proportions.^46^ In fact, it has been reported that the selection of the signature matrix often influences results more than the choice of the deconvolution algorithm itself.^12^ For CIBERSORTx, it is recommended that the signature matrix samples are preprocessed with the same steps as the samples to be deconvoluted.^9^ Various parameters in the signature matrix creation can have a considerable impact on the deconvolution performance. The minimum and maximum number of proteins (or genes) per cell type is one such parameter, which is investigated in Figure 6. Notably, setting this threshold too high results in considerably worse deconvolution performance.

**Figure 6:**
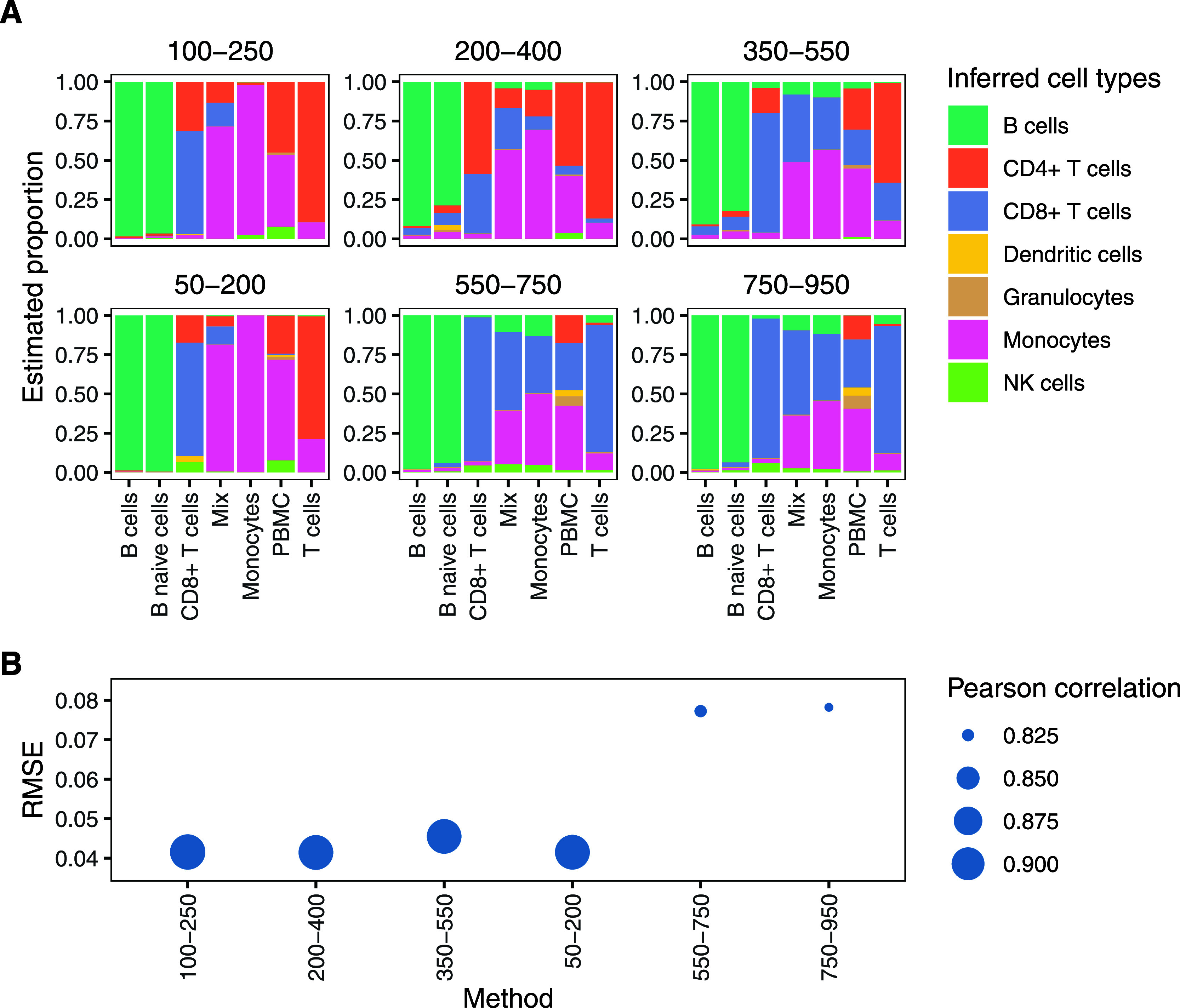
Influence of signature matrix creation parameters on deconvolution of DIA-based immune cell samples, using the Rieckmann reference proteome. Labels indicate the minimum and maximum number of proteins per cell type to consider. (A) Estimated cell-type proportions with different ranges of minimum and maximum number of proteins to consider per cell type. (B) The corresponding RMSE and Pearson correlation for simulated sample mixtures generated from the Rieckmann et al. data with the same signature matrices.

### Validation of Simulation Methodology

As some of the benchmarks are based on the simulation of samples, we also performed a validation of the accuracy of this methodology. By comparing the deconvolution results from a mixed sample consisting of equal proportions of CD8+ T cells and monocytes (in terms of cell count), we found that our simulation approach results in comparable outcomes to experimentally mixing samples (Figure 7). A shared limitation of both approaches is the presence of spillover between CD8+ and CD4+ T cells, suggesting that it remains difficult to distinguish between these subtypes with proteomics data. This effect has also been found to be widespread with transcriptomics data.^11^ The discrepancy between the deconvoluted proportions and the actual can probably be explained by the protein content bias. Monocytes are known to be larger than T cells and therefore contain more protein. For transcriptomics, the mRNA bias of different cell types has been taken into account with some algorithms such as EPIC.^10^ This represents one area of improvement for proteomics deconvolution algorithms, as no algorithm takes the protein content bias into account. This issue can potentially be partially mitigated by measuring and normalizing the injected sample amounts when performing the mass spectrometry analysis.

**Figure 7:**
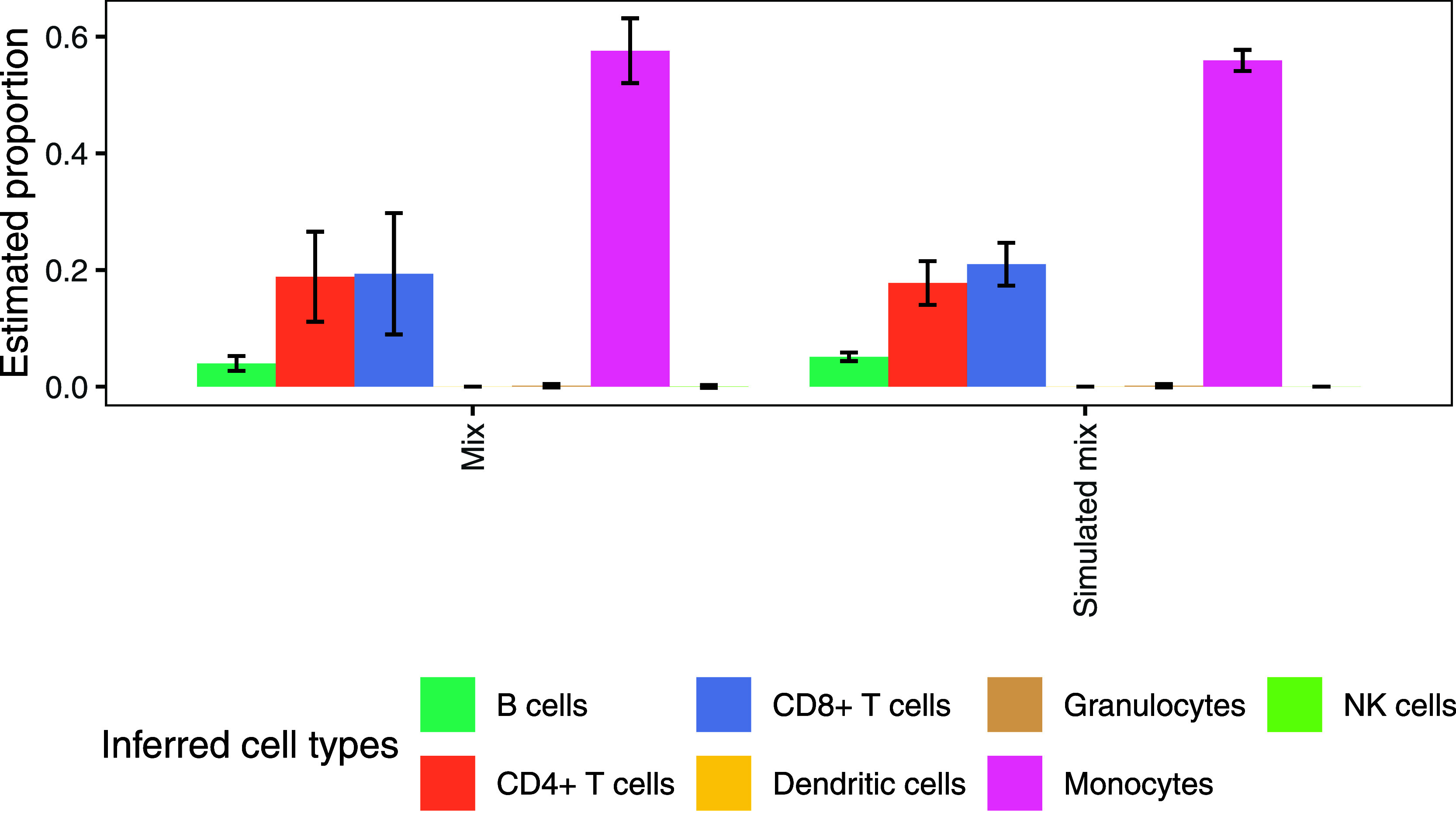
Validation of the in silico simulation approach against real 50–50 mixtures of CD8+ T cells and monocytes in DIA-based proteomics data. Four replicates of the real mix are compared to 100 simulated mixtures, both deconvoluted using CIBERSORT with the Rieckmann-derived signature matrix. Error bars represent standard deviations across replicates or simulations.

### proteoDeconv R Package

We developed the R package proteoDeconv to streamline the use of proteomics data for immune cell deconvolution. To the best of our knowledge, proteoDeconv is the only R package specifically designed for cell-type deconvolution using proteomics data. While there are related tools, such as the benchmarking platform Decomprolute,^20^ its focus is primarily on performance evaluation, offering metrics like correlation with transcriptomic data from CPTAC. However, Decomprolute does not assess the impact of different preprocessing strategies, which can significantly influence deconvolution outcomes in proteomics workflows. Other deconvolution frameworks include immunedeconv^11^ and omnideconv.^47^ However, these packages are not directly compatible with proteomics data, as it requires specific preprocessing steps. The ability to manage these preprocessing requirements is a distinctive feature of proteoDeconv.

## Discussion

As the end products of the central dogma, proteins offer a direct representation of cellular function, potentially providing more accurate estimates of cell type composition than transcriptomics-based approaches. In this study, we explored the feasibility of proteomics-based immune cell deconvolution by generating mass spectrometry data from purified immune cell populations, alongside simulated mixtures designed to model complex samples. By comparing estimated cell-type proportions against known compositions, evaluated through metrics like RMSE and correlation coefficients, we aimed to uncover factors that influence deconvolution performance in a proteomics context.

When comparing our results to transcriptomics-based benchmarks, it is notable that the Pearson correlations observed for our simulated proteomics data fall within a similar range (∼0.7–0.9) as those reported for well-matched reference matrices in transcriptomics studies.^12^ This suggests that proteomics-based deconvolution can achieve comparable performance, supporting its feasibility as an alternative or complementary approach to transcriptomics for studying immune cell composition.

One of the observations from our analyses is the strong influence of data quality and proteomic depth on deconvolution performance. We found that when the signature matrix and the samples being deconvoluted originate from the same data set—a controlled, albeit unrealistic scenario—the average Pearson correlation reached as high as 0.94. This reflects an ideal case where technical variability is minimized, but it also highlights the importance of using high-quality data with comprehensive proteomic coverage.

Another important aspect is the handling of missing values, which is a common challenge in proteomics. While imputation is often necessary, particularly in data-dependent acquisition (DDA) workflows, its impact on deconvolution performance can be complex. Our findings suggest that conservative imputation strategies, such as minimum-value imputation, tend to improve deconvolution performance more effectively than methods like Random Forest imputation or k-nearest neighbors (kNN) imputation. This pattern was observed across both our own data and the Rieckmann data set, indicating that conservative approaches may be generally preferable in the context of deconvolution.

The choice of signature matrix also plays an important role in deconvolution outcomes. When comparing proteome-derived and transcriptome-derived signature matrices, we observed that the proteome-derived matrices generally provided better performance when applied to proteomics data. This aligns with findings from previous studies^20^ and is consistent with the moderate correlation (∼0.4) typically observed between mRNA and protein abundance.^45^

Regarding the choice of using a reference-based or marker-based deconvolution algorithm, it appears that the reference-based methods can achieve superior performance. As was found by Avila Cobos et al.,^12^ reference-based methods generally perform better than marker-based methods with transcriptomics data. However, Feng et al.^20^ found that the reference-based methods benchmarked in their study resulted in worse deconvolution performance. Petralia et al.^18^ also argues that marker-gene-based methods are more suitable for proteomics data since reliable reference data are difficult to find. Theoretically, the choice of deconvolution method—whether it is marker-based or reference-based—has an impact on the deconvolution performance. For proteome data, the reduced depth in terms of number of measured proteins/genes compared to transcriptomics is a factor that may make marker-based methods less appropriate if the specific markers are not detected. A potential problem with reference-based methods is that changes in protein expressions can alter the conditions for deconvolution when less specific proteins are used in the signature.

While the motivation for this study is to eventually be able to reliably deconvolute immune cell composition of proteome samples, the samples that have been investigated are derived from PBMCs. The proteome of immune cells in PBMCs and tissue samples can be expected to vary, and including diseased samples in the signature matrix for transcriptome deconvolution has been shown to increase deconvolution accuracy and reduce biological bias.^46^ Still, many transcriptomics signatures are based on PBMCs and are very effective also for tumor sample deconvolution.^8^

This work represents an early step toward establishing robust methods for cell-type deconvolution with proteomics data. It is clear that gold-standard data sets, where the immune cell proportions are known, would enable further validation and method development. Furthermore, single-cell proteomics holds potential for refining signature matrix construction, potentially offering increased granularity and enhancing the accuracy of proteomics-based cell-type deconvolution.

## Conclusions

In conclusion, our study shows that proteomics offers a promising data source for estimating cell type compositions. By analyzing mass spectrometry proteome data and simulated immune cell mixtures, we recommend using high-quality, high-depth proteomic data for both sample and signature matrix construction. Additionally, employing conservative imputation methods, specifically minimum-value imputation, is important to improve deconvolution accuracy. A proteome-based reference matrix outperforms a transcriptome-based one, and algorithmically the reference-based methods appear to be most suitable for deconvolution. These insights highlight the feasibility of proteomics data in determining cellular compositions.

## Supplementary Material





## Data Availability

The mass spectrometry proteomics data have been deposited to the ProteomeXchange Consortium via the PRIDE^30^ partner repository with the data set identifier PXD056050.

## Notes

Figure S1: Deconvolution results comparing methods for handling duplicate proteins and protein groups (PDF)
Table S1: Performance metrics for simulated mixtures with different deconvolution algorithms and preprocessing steps (XLSX)
