# Supplementary material for: Considerations and Software for Successful Immune Cell Deconvolution Using Proteomics Data
Source: J Proteome Res. 2025 Jul 14;24(8):3751–61. doi: 10.1021/acs.jproteome.4c00868 (PMC12322953; doi:10.1021/acs.jproteome.4c00868)
Supplement: Supplementary file 1 [file pr4c00868_si_001.pdf]

Supporting Information: Considerations and Software for Successful Immune Cell Deconvolution using Proteomics Data

Måns Zamore,† Sergio Mosquim Junior,† Sebastian L Andree,† Can Altunbulakli,† Malin Lindstedt,† and Fredrik Levander*,†,‡

† *Department of Immunotechnology, Lund University, SE-22363, Lund, Sweden,*

‡ *National Bioinformatics Infrastructure Sweden, Science for Life Laboratory, Lund University, SE-22363, Lund, Sweden,*

Table of contents

Page

Figure S1 **S2**

Table S1 **(xlsx)**

Figure S1

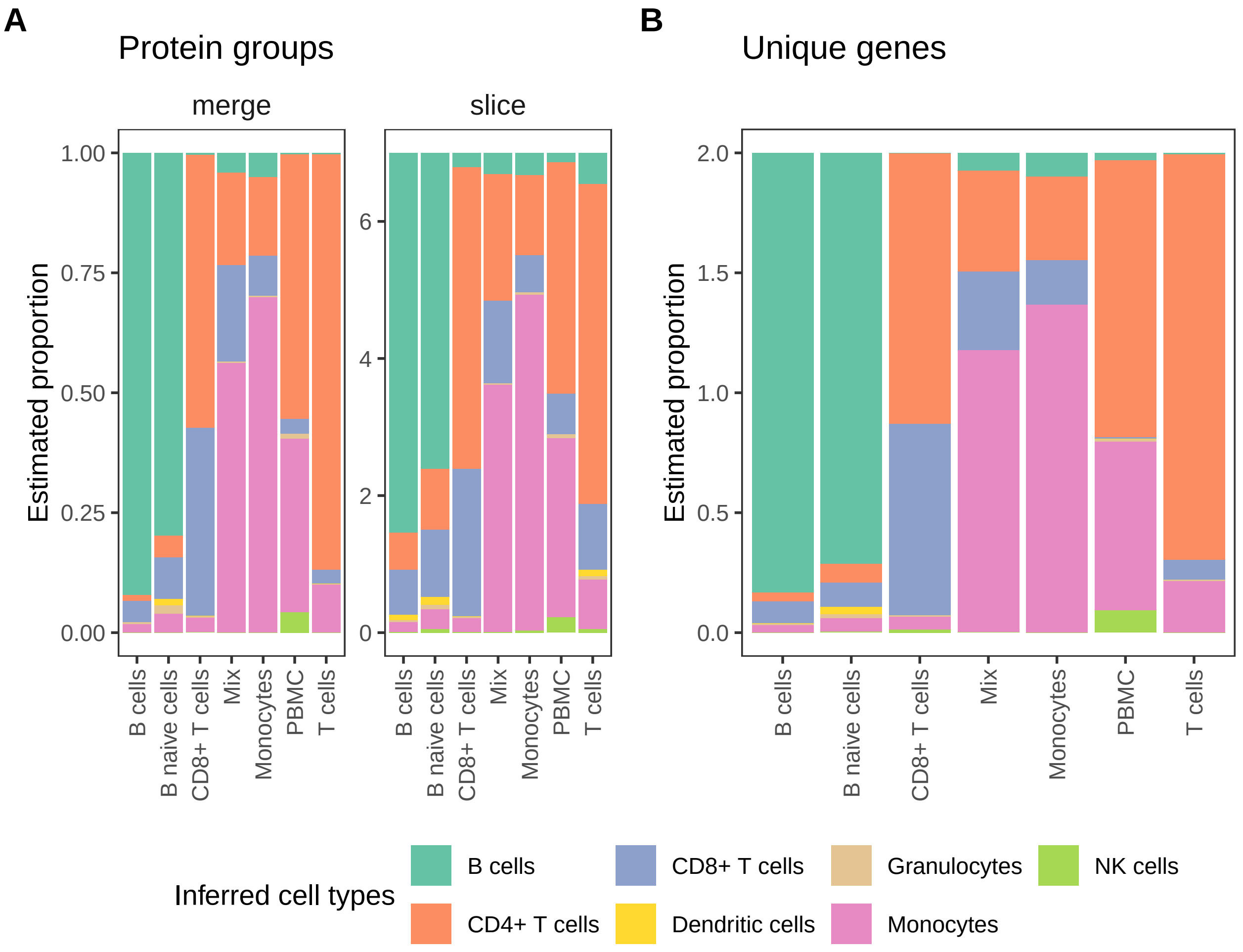

Figure S1. Deconvolution outputs from CIBERSORT on DIA-based immune cell data (pure cell types, a 50–50 CD8+ T/monocyte mix, and PBMC) using a signature matrix derived from the Rieckmann et al. immune cell reference proteome. (A) Comparison of merging intensities of duplicate proteins versus selecting (“slicing”) the row with the highest median intensity, using the protein groups file from DIA-NN. (B) Deconvolution results using the unique genes matrix from DIA-NN, where merging and slicing yield identical results since no duplicate genes are present.
